# Supplementary material for: Identification of photoperiod-induced specific miRNAs in the adrenal glands of Sunite sheep (Ovis aries)
Source: Front Vet Sci. 2022 Jul 22;9:888207. doi: 10.3389/fvets.2022.888207 (PMC9354845; doi:10.3389/fvets.2022.888207)
Supplement: Supplementary file 1 [file Data_Sheet_1.ZIP › Supplementary file/miRDeep2 file.zip!/miRDeep2 file/Know_Structure/SP42A2/oar-mir-376c.pdf]

miRBase precursor : oar-mir-376c
Total read count : 543
oar-miR-376c-5p read count : 228
oar-miR-376c-3p read count : 315
remaining reads : 0

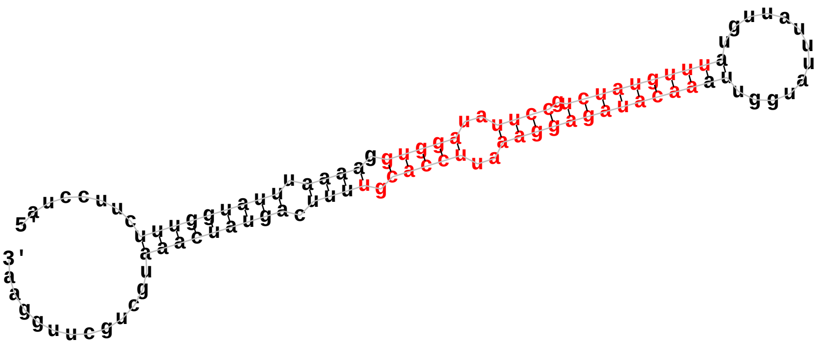

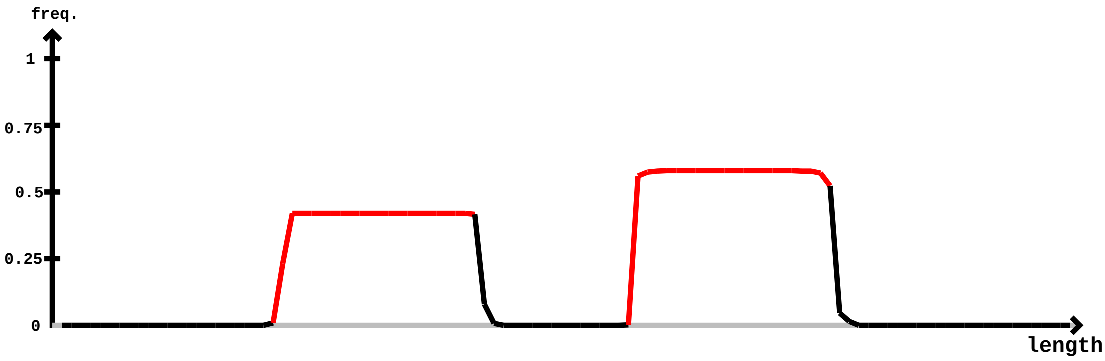

oar-miR-376c-3p

oar-miR-376c-5p

```
5'- auccuucuuugguauuuaaaagguggauauuccgucuauguuuauguuauuuaugguuaaacauagaggaaauuccacguuuucaguaucaaaugcugcuuggaa   -3'   exp
    .......((((((((.((((.(((((..((((.((((((((((............))))))))))))))..))))).)))).))))))))...............    reads      mm         sample
    .....................gguggauauuccgucuauguuu..............................................................    5          0          seq
    ......................guggauauuccgucuaugu................................................................    2          0          seq
    ......................guggauauuccgucuauguuu..............................................................    96         0          seq
    ......................guggauauuccgucuauguuua.............................................................    21         0          seq
    ......................guggauauuccgucuauguuuau...........................................................    2          0          seq
    .......................uggauauuccgucuauguuu..............................................................    82         0          seq
    .......................uggauauuccgucuauguuua.............................................................    18         0          seq
    .......................uggauauuccgucuauguuuau...........................................................    2          0          seq
    ..........................................................aaacauagaggaaauuccacgu.........................    1          0          seq
    ...........................................................aacauagaggaaauucc.............................    1          0          seq
    ...........................................................aacauagaggaaauuccac...........................    4          0          seq
    ...........................................................aacauagaggaaauuccacg..........................    26         0          seq
    ...........................................................aacauagaggaaauuccacgu.........................    257        0          seq
    ...........................................................aacauagaggaaauuccacguu........................    13         0          seq
    ...........................................................aacauagaggaaauuccacguuu.......................    2          0          seq
    ............................................................acauagaggaaauuccacguu........................    4          0          seq
    ............................................................acauagaggaaauuccacguuu.......................    4          0          seq
    .............................................................cauagaggaaauuccacguuu.......................    2          0          seq
    ..............................................................auagaggaaauuccacgu.........................    1          0          seq
```
